# Reanalysis and validation of the transcriptional pleural fluid signature in pleural tuberculosis

**DOI:** 10.3389/fimmu.2023.1256558

**Published:** 2024-01-15

**Authors:** Raquel da Silva Corrêa, Thyago Leal-Calvo, Thiago Thomaz Mafort, Ana Paula Santos, Janaína Leung, Roberta Olmo Pinheiro, Rogério Rufino, Milton Ozório Moraes, Luciana Silva Rodrigues

**Affiliations:** ^1^ Laboratory of Immunopathology, Medical Sciences Faculty, Rio de Janeiro State University (FCM/UERJ), Rio de Janeiro, Brazil; ^2^ Laboratory of Leprosy, Oswaldo Cruz Institute, Oswaldo Cruz Foundation (IOC/FIOCRUZ), Rio de Janeiro, Brazil; ^3^ Department of Pulmonary Care, Pedro Ernesto University Hospital, Rio de Janeiro State University (HUPE/UERJ), Rio de Janeiro, Brazil

**Keywords:** tuberculosis, pleural tuberculosis, exudative effusion, gene expression, *Mycobacterium tuberculosis*

## Abstract

**Introduction:**

Pleural tuberculosis (PlTB), the most common site of extrapulmonary TB, is characterized by a paucibacillary nature and a compartmentalized inflammatory response in the pleural cavity, both of which make diagnosis and management extremely challenging. Although transcriptional signatures for pulmonary TB have already been described, data obtained by using this approach for extrapulmonary tuberculosis and, specifically, for pleural tuberculosis are scarce and heterogeneous. In the present study, a set of candidate genes previously described in pulmonary TB was evaluated to identify and validate a transcriptional signature in clinical samples from a Brazilian cohort of PlTB patients and those with other exudative causes of pleural effusion.

**Methods:**

As a first step, target genes were selected by a random forest algorithm with recursive feature elimination (RFE) from public microarray datasets. Then, peripheral blood (PB) and pleural fluid (PF) samples from recruited patients presenting exudative pleural effusion were collected during the thoracentesis procedure. Transcriptional analysis of the selected top 10 genes was performed by quantitative RT-PCR (RT-qPCR).

**Results:**

Reanalysis of the public datasets identified a set of candidate genes (*CARD17, BHLHE40, FCGR1A, BATF2, STAT1, BTN3A1, ANKRD22, C1QB, GBP2*, and *SEPTIN4*) that demonstrated a global accuracy of 89.5% in discriminating pulmonary TB cases from other respiratory diseases. Our validation cohort consisted of PlTB (*n* = 35) patients and non-TB (*n* = 34) ones. The gene expressions of *CARD17*, *GBP2*, and *C1QB* in PF at diagnosis were significantly different between the two (PlTB and non-TB) groups (*p* < 0.0001). It was observed that the gene expressions of *CARD17* and *GBP2* were higher in PlTB PF than in non-TB patients. *C1QB* showed the opposite behavior, being higher in the non-TB PF. After anti-TB therapy, however, *GBP2* gene expression was significantly reduced in PlTB patients (*p* < 0.001). Finally, the accuracy of the three above-cited highlighted genes in the PF was analyzed, showing AUCs of 91%, 90%, and 85%, respectively. *GBP2* was above 80% (sensitivity = 0.89/specificity = 0.81), and *CARD17* showed significant specificity (Se = 0.69/Sp = 0.95) in its capacity to discriminate the groups.

**Conclusion:**

*CARD17*, *GBP2*, and *C1QB* showed promise in discriminating PlTB from other causes of exudative pleural effusion by providing accurate diagnoses, thus accelerating the initiation of anti-TB therapy.

## 1 Introduction

It is estimated that, per annum, there are roughly 10 million people with active tuberculosis (TB), and 1.3 million subsequent deaths from TB worldwide, being the world’s second leading cause of death from a single infectious agent after coronavirus disease (COVID-19) (1). Brazil is among the 30 countries with the highest TB rates, registering an annual estimated 80,000 new cases at an incidence of 36.3 cases/100,000 inhabitants and nearly 4,500 deaths (2). In 2021, the State of Rio de Janeiro, where this study was conducted, showed a disease incidence rate of 67.4/100,000 inhabitants, occupying the second position in Brazil (3).

Tuberculosis diagnosis and management remain a challenge due to the enormously complex and intricated immunopathology involved in combating *Mycobacterium tuberculosis* (Mtb), specifically in the extrapulmonary manifestations of the disease such as pleural TB (PlTB)—the most common site of extrapulmonary TB. PlTB is typically characterized by unilateral pleural effusion, pleuritic chest pain, persistent coughing, fever, nocturnal sweats, dyspnea, and weight loss (4, 5). The laboratory diagnosis of PlTB may be facilitated by the thoracentesis procedure by providing highly valuable clinical samples such as of exudative pleural fluid (PF), whose microbiological, biochemical, and immunological aspects can be analyzed. The compartmentalized immune response against Mtb in the pleural cavity seems to be paucibacillary, being enriched by a cytokine milieu that favors a lymphocytic dominant T-helper 1 (Th1), which is responsible for producing high levels of interferon-gamma (IFN-γ) and other Th1 cytokines (6–9). In addition, histological examination of pleural biopsies identifying caseating granulomas or visualization of acid-fast bacilli in the tissue and/or high levels of adenosine deaminase (ADA) in PF are also relevant guides toward a tuberculosis diagnosis (4). A fuller understanding of this compartmentalized and dynamic immune response may lead to a better comprehension of the varied antimycobacterial responses at different TB infection sites as well as contribute to a more timely and accurate diagnosis.

Among the *omics* approaches, whole-blood transcriptomics analysis has particularly contributed to gene profile identification in pulmonary TB and to a broader understanding of the mechanisms involved in the immune response and pathogenesis of many infectious diseases (10–12) in its ability to discriminate between active versus latent TB and compare characteristics of healthy uninfected individuals to those with pulmonary diseases (13–17). Altogether, these transcriptomic studies revealed a differential gene expression mainly represented by interferon-inducible pathways and pathogen/antigen recognition receptor-blood signatures. Subsequent works have evaluated the cytokine gene expression profile in samples from the pleural effusion of patients diagnosed with TB and diseases of other etiologies using blood (18, 19) and PF (20, 21). However, the transcriptomic analyses of extrapulmonary TB, particularly in PlTB, that use PF are scarce and provide limited heterogeneous data since most only utilize blood.

In the present study, signature genes that are differentially expressed in patients with pleural TB compared to other exudative etiologies that lead to pleural effusion were investigated. For this purpose, a preliminary reanalysis of public transcriptional datasets relative to pulmonary TB from Bloom et al. (2013) (22) was conducted. The top 10 candidate genes were identified and validated in paired PF and whole-blood samples collected at diagnosis and after anti-TB therapy. It is our hope that the data obtained in the present study contributes to the identification of new diagnostic and therapeutic procedures aiming to profoundly impact the management of PlTB patients.

## 2 Materials and methods

### 2.1 Clinical cohort and ethics statement

**Figure 1:**
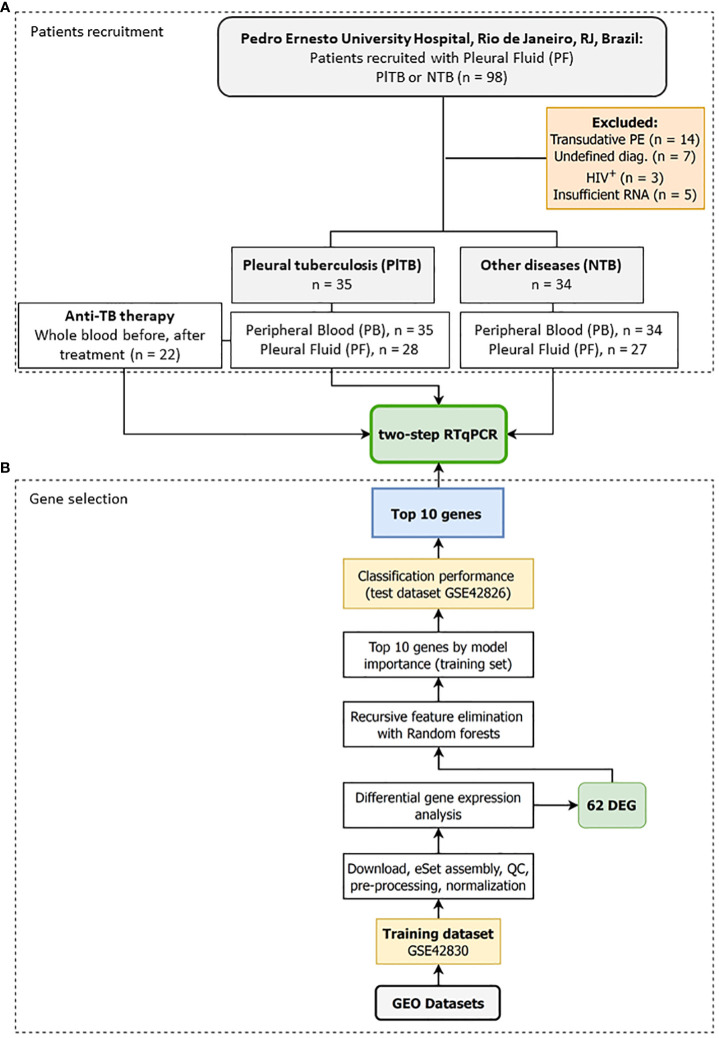
Study design to identify a transcriptomic signature associated with pleural tuberculosis. The study was divided into two stages: **(A)** Step A—Patients recruitment: Pleural fluid and whole blood were collected from patients showing pleural effusion with an indication for thoracentesis followed by grouping the participants into either pleural tuberculosis (PlTB) or non-tuberculosis (non-TB). A new whole-blood sample was collected from PlTB patients after anti-TB treatment. **(B)** Step B—Gene selection: *In silico* analysis was performed to define the top 10 genes differentially expressed in the whole blood from pulmonary tuberculosis patients based on the aforementioned study by Bloom et al. (22), and applied to the pleural effusion validation cohort by reverse transcription quantitative PCR (RT-qPCR).

A longitudinal study was conducted with both male and female patients over the age of 18 suspected of pleural effusion, for whom an indication for thoracentesis was warranted, in attendance between June 2015 and February 2020 at the Pulmonary and Tisiology Service—a tertiary care center in the City of Rio de Janeiro, RJ, Brazil—in the Pedro Ernesto University Hospital of the Rio de Janeiro State University (HUPE/UERJ). The patients who were under 18, pregnant, or refused consent were not recruited. Among the 98 recruited patients, 29 were excluded: 14 had transudative pleural effusion (cardiac or renal failure), 7 had an undefined diagnosis, 3 were HIV seropositive, and 5 had an insufficient RNA sample (**Figure 1A**). PF and peripheral blood (PB) samples were collected before treatment. Only PlTB patients had a new blood collection at the end of their anti-TB treatment.

The study protocol was approved by the HUPE/UERJ Ethics Committee (approval number 1,100,772), closely following the recommendations of the Helsinki Declaration. Participants were properly informed about the study objectives, and all voluntarily signed the written informed consent form prior to enrollment and sample collection. Medical information was obtained from electronic records and additional survey questionnaires.

### 2.2 Reanalysis and definition of candidate genes from public datasets

Because there are no available transcriptomic datasets of pleural effusion from tuberculosis, we reanalyzed two blood datasets published by Bloom et al. (2013) (22) and available in GEO (GSE42830 and GSE42826) to select genes that classify pulmonary TB from lung cancer, a common differential diagnosis (**Figure 1B**). The background-subtracted data were downloaded from GEO and quantile normalized using routines from the preprocessCore package v.1.56.0. Microarray probes were reannotated using the illuminaHumanv4.db annotation v.1.26 and AnnotationDbi packages v.1.56.2. Duplicated ENTREZIDs were removed while maintaining the probe with the greatest average expression across all samples. Differential gene expression analysis was then performed by fitting gene-wise linear models via limma v.3.50.0, making adjustments for biological sex and ethnic categorical variables (23), and comparatively testing tuberculosis (*n* = 16) vs. lung cancer (*n* = 8). After selecting the differentially expressed genes, they were ranked by model importance using Recursive feature elimination (RFE) with the random forests algorithm implemented in the caret package v.6.0-90. Briefly, the normalized log_2_ gene expression matrix from GSE42830 was adjusted to remove the estimated effects of sex and ethnicity using limma’s RemoveBatchEffect function. Then, only the differentially expressed genes |log_2_FC| ≥ 1 and FDR ≤ 0.01 were used for feature selection. RFE with random forests was set to retain 2-to-20 genes by way of repeated 10-fold cross-validation with 50 repetitions by maximizing the area under the curve (AUC) metric. The variable importance output was used to rank and extract the top 16 genes from the RFE. Finally, the accuracy of these 16 genes was tested using a subset of GSE42826, the second independent test dataset, and analyzed as previously described. The variable importance of the RFE on the training dataset and the AUC together with their sensitivity (Se) and specificity (Sp) on the test set (TB, *n* = 11; lung cancer, *n* = 8) was used to select the final 10 gene list for RT-qPCR replication with our cohort from Rio de Janeiro, RJ, Brazil (**Figure 1**).

### 2.3 Diagnostic criteria

The diagnoses were made by specialized physicians from the Pulmonology and Tisiology Service at HUPE/UERJ through the analysis of radiological, tomographic, cytological, histological, and microbiological exams (bacilloscopy together with PF and pleural biopsy cultures), and clinical epidemiological data. *PlTB* was defined as a result of a detailed physical examination and the existence of at least one diagnostic criterion: (i) Ziehl–Neelsen stain positivity, or isolation of Mtb in the respiratory specimen, PF, or pleural tissue. Other characteristics included the (ii) identification of granuloma formation in the histopathological analysis, (iii) clinical manifestations of PlTB (fever, pleuritic pain, dyspnea, coughing, nocturnal sweats, hyporexia, and/or weight loss), and (iv) lymphocytic and exudative pleural effusion in combination with an ADA dosage above 40 IU/L, showing full recovery after at least 6 months of anti-tuberculous treatment. *Non-tuberculosis (NTB)* cases were defined as those with pleural or pleuropulmonary diseases other than TB in which the diagnoses were based on clinical, laboratory, radiological, microbiological, and cytopathological/histopathological features. Patients who did not fit the criteria used for the PlTB diagnosis as described above and with unknown causes of pleural effusion were categorized as having undefined pleural effusion and were considered *non-TB*, as previously described by Lisboa and colleagues (6).

### 2.4 Sample collection and processing

Whole-blood and PF (2.5 mL) samples were collected into PAXGene tubes (QIAGEN, Germany) and stored at −20°C after stabilization at room temperature for 2 h. RNA was isolated using the PAXgene Blood RNA Kit (QIAGEN) and DNAse-treated as per instructions of the manufacturer. RNA was eluted into a final volume of 40 μL and stored at −80°C until further use. RNA quality was assessed by NanoDrop spectrophotometer and non-denaturing agarose gel electrophoresis. Non-degraded RNA was reverse transcribed into cDNA using the SuperScript III (Thermo-Fisher Scientific, USA) according to the instructions of the manufacturer. Finally, cDNA was diluted to a working concentration of 5 ng/μL with Tris-EDTA buffer (10 mM Tris, EDTA 0.1 mM) and kept at −20°C.

### 2.5 Reverse transcription quantitative PCR

Gene expression experiments were done using the Fast SYBR Green Master Mix (Thermo-Fisher Scientific, USA) in line with the instructions of the manufacturer. Briefly, a 10-μL final reaction contained 10 ng of cDNA, 300 nM of each primer (Thermo-Fisher Scientific, USA) (**Supplementary Table 1**), and 5 μL of SYBR Green Master Mix. Reactions were performed in duplicate in a Viia7 (Applied BioSystems, USA) machine using the default thermal cycling program that included the melting curve. Raw data were exported in an RDML format using QuantStudio v.1.3 software after assessing primer specificity from the melting curves. Then, efficiency-adjusted N_0_ values were obtained using LinRegPCR v.2021.2 with default parameters (24, 25). The N_0_ values for genes of interest were normalized by calculating the ratio to the geometric average of N_0_ values for two reference genes (*RPLP2* and *POLR2A*), followed by a log_2_ transformation. These logarithmized relative expression values were used for visualization and statistical inference.

### 2.6 Statistical analyses

Descriptive analyses of the study population, according to its sociodemographic and clinical characteristics among PlTB and NTB patients, were determined by the nonparametric Mann–Whitney test for continuous variables or Fisher’s exact test for comparison of the relative frequencies of categorical variables. Relative gene expression in log2 was used for comparing means between groups. Statistical analyses were done by fitting gene-wise linear mixed models with lme4 v.1.1.2 in R 4.1. Briefly, two models were used. The first estimated the effects between disease groups (TB, NTB) and tissues (PB, PF), i.e., both between- and within-subject effects, respectively. For this model, the mixed model (RML criterion) included three categorical variables such as fixed effects (“batch”, “diagnosis”, and “tissue”) and “patient id” as a random intercept. The second model included only the “period” variable (categorical within-patient) as fixed effects and “patient id” as a random intercept. Outliers were removed during model fitting by excluding observations with absolute-scaled Pearson standardized residuals greater than 2.5. Next, the estimated marginal means and specific contrasts were obtained using emmeans v.1.7.2. *p*-values and 95% confidence intervals for the TB-NTB × PB-PF contrasts (four estimates) were adjusted within genes via the Sidak method. Model estimates were plotted over the normalized data alongside confidence intervals. To estimate the effect of demographic and laboratory variables in gene expression, each variable was separately included in the model as a fixed effect, both as an additive or interaction term. Continuous laboratory variables were log_10 +_ 1 transformed before any analysis. Then, *p-* values for the coefficients of these covariates were extracted and adjusted according to the Benjamini–Hochberg (1995) (26) procedure to simultaneously control the false discovery rate across all genes simultaneously. This was done to limit the type I error rate due to the large number of estimated coefficients. Any covariate with an adjusted *p*-value ≤ 0.1 was further investigated by Spearman correlation analyses and graphs. ROC curves and AUC were obtained with the pROC package v.1.18.0, and the best threshold was chosen by Youden’s *J* statistic (1950) (27) and Delong’s 95% confidence interval. Heatmaps were drawn using ComplexHeatmap v.2.10.0 from the gene-wise scaled log_2_ data, and genes were clustered using Spearman’s *rho* as the distance metric.

## 3 Results

### 3.1 Bioinformatics reanalysis of a public microarray datasets from pulmonary tuberculosis patients

For the test dataset, GSE42830, the differentially expressed genes (DEGs) from tuberculosis vs. neoplasia were used in the recursive feature elimination (RFE) algorithm to select candidate genes from Bloom et al. (2013) (22). In this comparison, we found 120 DEGs with an adjusted *p*-value of (FDR) ≤ 0.01 e |log_2_| ≥ 1. These genes were then subjected to RFE analysis via the random forest algorithm. The top 16 genes were subsequently tested for their classification potential in the GER42826 test data set and ranked according to the importance of these genes in the classification of tuberculosis vs. neoplasia in these samples (**Supplementary Table 2** shows the 10 genes with their importance values). In this test dataset, these 10 genes showed an AUC of 89.5% to distinguish tuberculosis vs. neoplasia. Finally, the top 10 genes were selected from this ranking for independent validation by RT-qPCR in samples from a population in Rio de Janeiro, RJ, Brazil (**Supplementary Figure 1**). **Supplementary Table 2** lists the 10 best genes according to their importance: *CARD17, BHLHE40, FCGR1A, BATF2, BTN3A1, C1QB, ANKRD22, GBP2, STAT1*, and *SEPTIN4*, in addition to their AUC, specificity, and sensitivity rates.

### 3.2 Characterization of the pleural effusion cohort

Among the 69 eligible patients in the present study, 41 (59.4%) were men and 28 (40.6%), were women, ranging in age from 18 to 92. In the PlTB group, the mean age was 40.7 and 60.9 in the non-TB group. Furthermore, 35 (50.7%) were diagnosed with PlTB (33 pleural tuberculosis, 2 pleuropulmonary) while 34 (49.3%) were classified as non-TB. At the beginning of the study, that is, prior to treatment and during diagnostic investigation, clinical samples (blood and PF) were collected at the moment of thoracentesis guided by transthoracic ultrasound. At another moment of the study, rather, at the end of anti-TB therapy, approximately 6 months later, PB samples were also collected solely from PlTB patients to evaluate the behavior of the genes of interest (**Figure 1A**). After treatment, as expected, pleural effusion disappeared and, as such, sample analysis from the pleural cavity was not possible.

**Table 1** shows the demographic, clinical, and biochemical characteristics of the PB and PF samples of the 69 patients with a confirmed diagnosis (PlTB = 35; NTB = 34). For the most part, the non-TB group consisted of patients with cancer, corresponding to more than 75% of the total [the main cancer types were adenocarcinoma (59.09%), lymphoma (4.5%), squamous carcinoma (4.5%), metastatic (9.09%), and undefined (22.72%)], followed by 11.8% with undefined diagnoses, 8.8% with non-tuberculous empyema, and 2.9% with systemic lupus erythematosus (SLE). In the PlTB group, more than 94% corresponded to cases affecting only the pleura; and 5.3% affected both the pleura and the lungs. All volunteers had respiratory symptoms (coughing and/or dyspnea and/or fatigue) and their chest x-rays, computed tomography, and/or ultrasonography findings show unilateral or bilateral pleural effusion associated or not with lung parenchymal changes. Among the pleural TB group, 71.4% of cases showed high and/or moderate pleural effusion complexity as ascertained by the pleural ultrasound results, in comparison to 41.2% of the NTB group, demonstrating a significant difference between the groups (*p*-value = 0.0155; **Table 1**).

**Table 1:** Characteristics of the study population.

Characteristics/Groups	Non-TB (*n* = 34)	Pleural TB (*n* = 35)	*p*-value
Age, mean (SD)	60.9 (16.8)	40.7 (18.9)	<0.001
Female (%)	16 (47.1%)	12 (34.3%)	0.332
Male (%)	18 (52.9%)	23 (65.7%)	
Diagnosis, *n* (%)			
Cancer	26 (76.5%)	0 (0%)	<0.001
Undefined diagnoses	4 (11.8%)	0 (0%)	
Non-tuberculous empyema	3 (8.8%)	0 (0%)	
Systemic lupus erythematosus	1 (2.9%)	0 (0%)	
Pleural TB	0 (0%)	33 (94.3%)	
Pleuropulmonary TB	0 (0%)	2 (5.3%)	
Pleural fluid categories by pleural cavity ultrasound, *n* (%)			
Low complexity	20 (58.8%)	10 (28.6%)	0.0155
Moderate/high complexity	14 (41.2%)	25 (71.4%)	
ADA, U/L			
Mean (SD)	19.6 (26.3)	55.4 (26.8)	<0.001
Positive ≥ 40 (%)	8.8%	71.4%	
Mononuclear cells, %			
Mean (SD)	77.8 (21.1)	89.3 (16.6)	0.0076
Polymorphonuclear cells, %			
Mean (SD)	22.2 (21.1)	12.6 (22.5)	0.00899
LDH, IU/L			
Mean (SD)	832 (1,650)	442 (490)	0.84
Mycobacteria culture			
Negative, %	100%	77.2%	0.229
AFB			
Negative, %	100%	82.8%	1
Pleural histopathology, %			
Granuloma with necrosis	0%	14.3%	<0.001
Granuloma without necrosis	0%	14.3%	
Nonspecific inflammatory infiltrate	32.4%	20%	
Malignant identification	35.2%	0%	
Missing	32.4%	51.4%	

ADA, adenosine deaminase; AFB, acid-fast bacillus; LDH, lactate dehydrogenase; SD, standard deviation; TB, tuberculosis. Pleural effusion categories were classified as either “low complexity” (homogeneous and/or anechoic) or “moderate/high complexity” (nonloculated, or complex loculated pleural effusion, respectively) by pleural cavity ultrasound.

Demographic, clinical, and biochemical characteristics of the pleural fluid of non-TB (n = 34) and TB patients (n = 35).

**Table 1** also depicts laboratory characteristics. ADA measurement was 71.4% positive (≥40 U/L) in PlTB patients compared to 7.7% in the non-TB ones (*p* < 0.001). Mononuclear cells were significantly higher in the PlTB group (*p* = 0.0076) while polymorphonuclear cells increased in the non-TB group (*p* = 0.0089). The negativity percentages in the Mtb and AFB culture tests in the PlTB group were very high, 77.2% and 82.8%, respectively. Lastly, in the histopathological examination, the presence of granuloma with necrosis was revealed to cover 14.3% of all PlTB cases.

### 3.3 Validation of the top 10 candidate genes in clinical samples from the pleural effusion cohort

The previously defined top 10 genes as a result of *in silico* analysis were validated by RT-qPCR assays in whole-blood and PF clinical samples taken from our Brazilian cohort. The *SEPTIN4* gene did not obtain detectable levels of messenger RNA (data not shown). The following nine genes were then analyzed by RT-qPCR assays: *CARD17*, *BHLHE40, FCGR1A, BATF2, BTN3A1, C1QB, ANKRD22, GBP2*, and *STAT1*.

**Figure 2:**
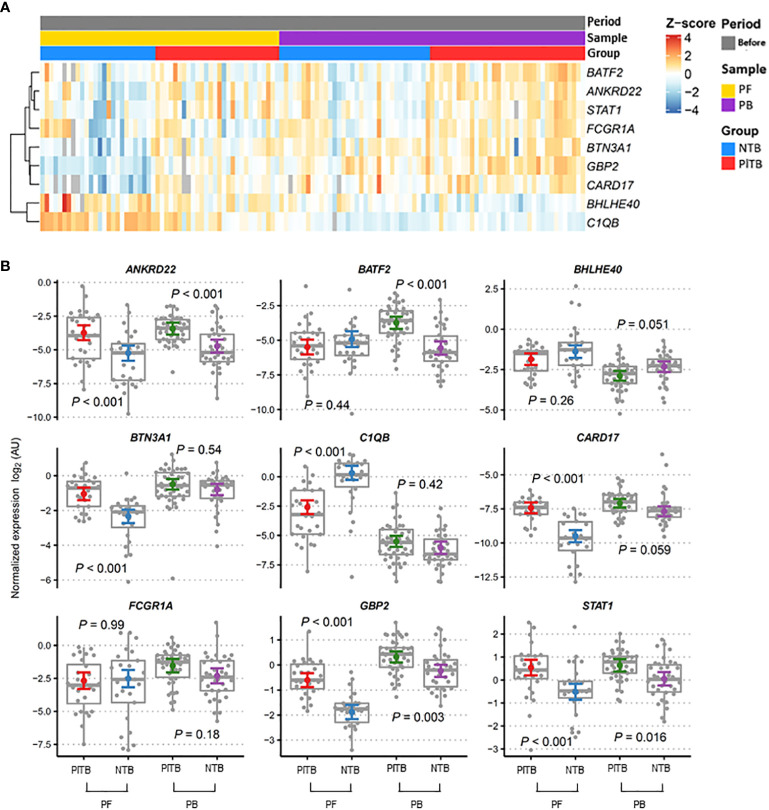
Blood and pleural fluid transcriptional signature associated with pleural tuberculosis. **(A)** Heatmap showing the relative expression of the pre-selected top 10 genes was analyzed by RT-qPCR from the study groups (red—PITB, pleural tuberculosis; blue—NTB, non-tuberculosis) and samples (yellow—PF, pleural fluid; purple—PB, peripheral blood) were obtained before anti-TB therapy (gray). Genes in rows were clustered using the Pearson correlation coefficient distance and the complete agglomeration method. **(B)** Gene expression profiles in the peripheral blood (PB) and pleural fluid (PF) of pleural tuberculosis (PlTB) and non-tuberculosis patients (NTB) were analyzed. The Tukey box graphs and dots showed normalized gene expression values while the colored dot and error bar displayed the linear model point estimates and their 95% confidence intervals, respectively. *p*-values were calculated from the mixed linear models for specifically planned comparisons and adjusted via the Sidak method per gene. AU, arbitrary units.

Transcriptional profiles of PlTB (red) and other non-TB (blue) etiologies of pleural effusion at diagnosis were clustered by whole blood (purple) and PF (yellow) in the heatmap plots and boxplots shown in **Figure 2**. The *Z* score demonstrated the behavior of each gene of interest regarding its up-or-down expression (from a bluish to a reddish color) in both groups as evidenced in the analyzed sample. In **Figure 2B** (boxplots), there are the same genes of interest with gene expression values normalized in log2. There was a significant difference among the gene expressions observed in the PlTB patients in comparison to the non-TB ones: *ANKRD22, BTN3A1, CARD17, GBP2*, and STAT1, all of which had *p*-values < 0.001. The *C1QB* gene, on the other hand, obtained a significantly higher gene expression in the PF in the non-TB group when compared to the PlTB one. The genes that showed a significant difference between groups, with higher values in the PB samples within the P1TB group, were *ANKRD22* (*p* < 0.001), *BATF2* (*p* < 0.001), *GBP2* (*p* = 0.003), and *STAT1* (*p* = 0.016). The expression of mRNA in the latter two genes (*GBP2* and *STAT1*) in both clinical samples was higher in the PlTB group. However, it is noteworthy that the distinction between the groups was more pronounced in the PF. As for the *ANKR22* gene, in both samples, there was a distinction between the groups (*p*-value < 0.001).

### 3.4 Expression of genes of interest after treatment with anti-TB therapy

**Figure 3:**
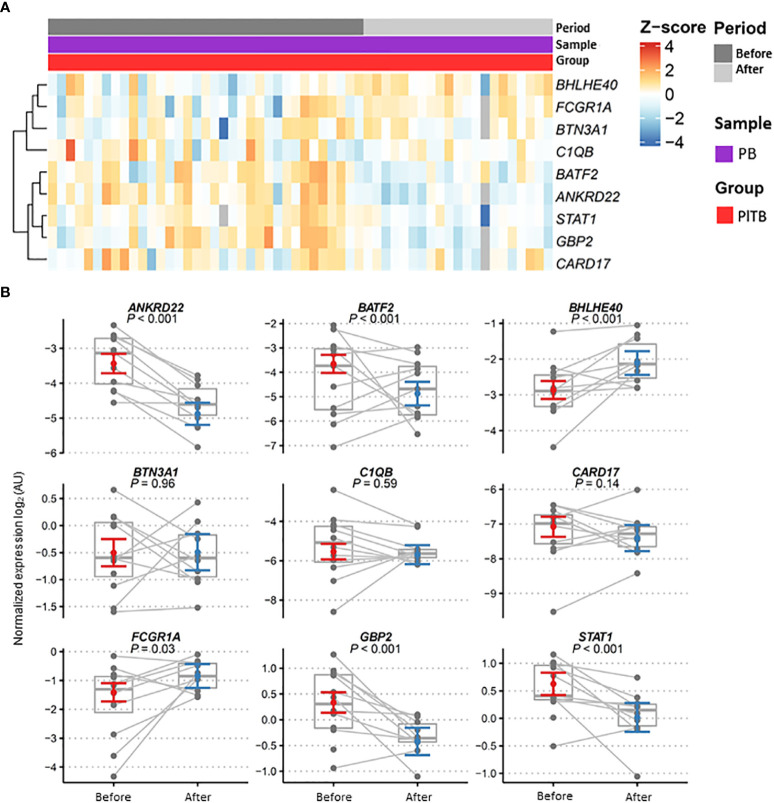
Gene expression profiles in patient blood after anti-TB therapy. **(A)** Heatmap showing the relative expression of the pre-selected top 10 genes analyzed by RT-qPCR from the pleural tuberculosis patients (PlTB, in red) and in the peripheral blood samples (PB, in purple) obtained before (dark gray) and after (light gray) anti-TB therapy. Genes in rows were clustered using the Pearson correlation coefficient distance and complete agglomeration methods. **(B)** Gene expression profiles in the blood of pleural tuberculosis patients before and after anti-TB therapy. Dots for these same patients are connected across periods. Model estimates and confidence intervals were obtained from linear mixed models after removing outliers (see Methods). Data are shown as normalized gene expressions (gray dots and box) and estimates based on linear mixed models (colored dot = marginal mean, error bars = 95% CI). AU, arbitrary units.

The longitudinal variation of gene expression in the PlTB patients (*n* = 12) after treatment with anti-TB therapy (**Figure 3**) was subsequently investigated. The heatmap graphs (**Figure 3A**) and the dot graph (**Figure 3B**), both representing two moments of the study with the blood-paired samples, were used. **Figure 3B** shows a gray, continuous line linking the expression of a given gene before (red color) and after treatment (blue color) for better understanding. Significant *p*-values are shown in the graphs, comparing the biological samples at these two moments of collection. Genes that had significant *p*-values with reduced post-treatment gene expression were *ANKRD22* (*p* < 0.001), *BATF2* (*p* < 0.001), *GBP2* (*p* < 0.001), and *STAT1* (*p* < 0.001). The genes that showed significant *p*-values with increased gene expression after treatment were *BHLHE40* (*p* < 0.001) and *FCGR1A* (*p* = 0.03) (**Figure 3B**).

### 3.5 Performance of the top 10 tuberculosis signature genes in the blood and pleural fluid

**Figure 4:**
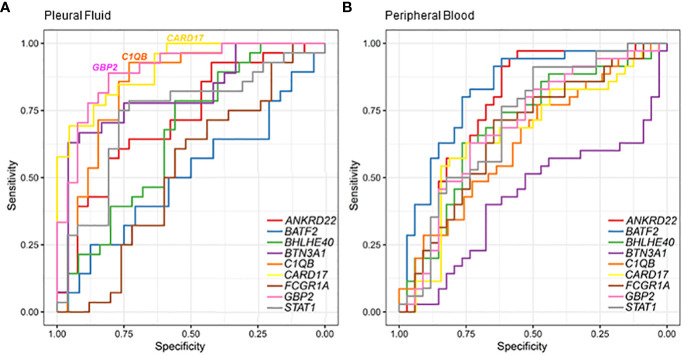
Classification of pleural tuberculosis using the top 10 gene expression profiles in the blood and pleural fluid along with the receiver operating characteristic (ROC) analysis of candidate genes validated by RT-qPCR to discriminate pleural tuberculosis from non-tuberculosis patients using pleural fluid **(A)** and peripheral blood **(B)**.

The receiver operating characteristic (ROC) analysis of the candidate genes in the RT-qPCR validation dataset (**Figure 4**) revealed the three most prominent genes in the PF sample: *CARD17, C1QB*, and *GBP2* (**Figure 4A**). Conversely, in the whole-blood samples, high values of sensitivity and specificity were not observed (**Figure 4B**). It is possible to better visualize these highlights in the PF samples of the sensitivity, specificity, and accuracy values in **Table 2**. In the PF, the *CARD17* gene had AUC, sensitivity, and specificity values of 0.91, 0.70, and 0.95, respectively; the *C1QB* gene had AUC, sensitivity, and specificity values of 0.84, 0.93 and 0.74, respectively. The *GBP2* gene had AUC, sensitivity, and specificity values of 0.90, 0.89, and 0.81, respectively (**Table 2**).

**Table 2:** Receiver operating characteristic (ROC) comparison of selected genes in discriminating pleural TB from non-TB diagnoses.

Sample	Gene	AUC [95% CI]	Specificity	Sensitivity	LR+	LR-
PF	*CARD17*	0.91 [0.83–0.99]	0.956522	0.703704	16.19	0.31
PF	*BATF2*	0.51 [0.35–0.67]	0.88	0.241379	2.01	0.86
PF	*STAT1*	0.73 [0.59–0.87]	0.740741	0.758621	2.93	0.33
PF	*BTN3A1*	0.81 [0.68–0.93]	0.92	0.678571	8.48	0.35
PF	*FCGR1A*	0.52 [0.36–0.69]	0.423077	0.724138	1.26	0.65
PF	*C1QB*	0.85 [0.74–0.96]	0.740741	0.931034	3.59	0.09
PF	*GBP2*	0.9 [0.82–0.98]	0.814815	0.892857	4.82	0.13
PF	*BHLHE40*	0.66 [0.51–0.81]	0.576923	0.793103	1.87	0.36
PF	*ANKRD22*	0.72 [0.58–0.86]	0.777778	0.586207	2.64	0.53
PB	*CARD17*	0.66 [0.52–0.8]	0.727273	0.657895	2.41	0.47
PB	*BATF2*	0.83 [0.73–0.93]	0.628571	0.921053	2.48	0.13
PB	*STAT1*	0.71 [0.58–0.83]	0.485714	0.864865	1.68	0.28
PB	*BTN3A1*	0.43 [0.29–0.57]	0.685714	0.421053	1.34	0.84
PB	*FCGR1A*	0.66 [0.53–0.8]	0.628571	0.684211	1.84	0.50
PB	*C1QB*	0.64 [0.5–0.77]	0.5	0.789474	1.58	0.42
PB	*GBP2*	0.69 [0.57–0.82]	0.714286	0.578947	2.03	0.59
PB	*BHLHE40*	0.69 [0.57–0.82]	0.771429	0.631579	2.76	0.48
PB	*ANKRD22*	0.77 [0.65–0.89]	0.628571	0.894737	2.41	0.17

Pleural fluid (PF), peripheral blood (PB), Gene performance (AUC sensitivity, specificity, and likelihood ratio). Highlighted in gray is the performance of the CARD17, C1QB, and GBP2 genes in pleural fluid samples. AUC, area under the curve; C, confidence interval; PB, peripheral blood; and PF, pleural fluid; LR+, likelihood positive ratio; LR−, negative likelihood ratio.

## 4 Discussion

Pleural tuberculosis is the main extrapulmonary clinical form of tuberculosis in which a reliable diagnosis can be reached by obtaining clinical samples by way of invasive procedures. In this context, a PlTB diagnosis remains a significant challenge, mainly due to the low bacillary load at the infectious site and the compartmentalized immune response (28, 29). The identification of new biomarkers associated with active disease may represent a paradigm shift in the clinical routine toward an early, quick, and accurate diagnosis. In the present study, we performed a bioinformatics reanalysis of previously published transcriptomic public datasets on pulmonary tuberculosis to validate the signature genes that are differentially expressed in PlTB patients when compared to other etiologies of exudative pleural effusion (lung neoplasms, pneumonia, autoimmune diseases, and non-tuberculous empyema). Our data revealed three genes—*CARD17*, *GBP2*, and *C1QB*—that demonstrated high accuracy in discriminating the PlTB from the non-TB patient groups.

Notable advances have been made in the development of the so-called “*omics*” sciences, namely, genomics, transcriptomics, proteomics, lipidomics, and metabolomics. Transcriptomic analysis in tuberculosis provided information regarding lung disease, contributing to the identification of transcriptional signatures, facilitating the ability to discriminate active from latent TB besides monitoring the treatment of the Mtb infection biomarkers in the development of active disease (30–33). However, it was our observation that data pertaining to the extrapulmonary forms were still scarce and heterogeneous. When beginning the present endeavor, a study by Bloom et al. (2013) (22) was selected from the published material in the literature to perform a reanalysis of the data using bioinformatics due to the common cellular immune pathways associated with both pulmonary tuberculosis and PlTB. Among the principal characteristics of the study by Bloom (22), it was clear that its experimental design was closer to our intentions regarding our cohort of patients in comparing pulmonary tuberculosis to other etiologies (pulmonary sarcoidosis, pneumonias, autoimmune diseases, and lung neoplasms).

The majority of transcriptomic studies have tracked transcriptional signatures in pulmonary tuberculosis while maintaining a systemic view in analyzing only whole-blood samples. Since PlTB, a disease with an immune and inflammatory response that is compartmentalized in the pleural space, was the focus of the present study, the site of infection is mandatory for this kind of exploration. In the study conducted by Bloom and colleagues (2013) (22), a small quantity of blood was obtained, similar to our samples. The importance of using a reduced volume is linked to greater short-term viability in the development of new point-of-care diagnostic tests (POCTs), which would be cost-effective and facilitate the rapid detection of diseases requiring immediate treatment (34–36).

In the present patient cohort, it was found that tuberculosis patients tended to be younger adults than those with other diseases (*p* < 0.001). The proportion of female and male patients was balanced in the non-TB group; (16/18) but, in the pleural TB group, there were almost twice as many men than women (12/23); data were also confirmed in other studies and detected by the World Health Organization (2, 37, 38). Moreover, the negativity in AFB and mycobacterial culture tests in PlTB was approximately 82% and 77%, respectively, reflecting the paucibacillary nature of PlTB (29, 39, 40). Granuloma with necrosis in the pleural TB group was present in only 14.3% of the samples, a percentage point below the value described in the literature of between 63% and 84% (40). In the end, the malignancy aspect in the non-TB group was found in 35.2% of the samples.

*CARD17* and *GBP2* were increased in the PlTB patient samples whereas *C1QB* was higher in the non-TB samples. These two identified genes could act as TB biomarkers. Hence, further research on their use should be encouraged. *CARD17* is a gene associated with mycobacterial infection. *CARD17*, upregulated in blood from resistant (MDR/RR)-TB when compared to the susceptible/mono-resistant TB drug (41), is part of a molecular gene signature that can discriminate active from latent TB (30).

Furthermore, *CARD17* is associated with the cellular response to the components of the bacterial wall and in the negative regulation of IL-1β. Caspase recruitment domain (CARD)-17 inhibits the release of IL-1β in response to LPS by monocytes. The assembly of inflammasomes is initiated upon activation of cytosolic pattern recognition receptors (PRRs), followed by polymerization of the pyrin domain (PYN)-containing and caspase recruitment domain (CARD)-containing proteins. CARD17 displayed crucial CARD interactions between caspase 1 protein through competitive binding and the amelioration of uric acid crystal-mediated NLRP3 inflammasome activation and inflammatory disease (42).

Guanylate-binding proteins (GBPs) are effector molecules involved in the important autonomic responses induced by pro-inflammatory stimuli, mainly IFNs (43). *GBP2*, induced by IFN-γ (44, 45), has been linked to a myriad of different cancer types as an oncogenic gene. In the present study, it was seen that *GBP2* expression was significantly higher in the PF of the tuberculosis compared to the non-tuberculosis group, as also found by Zak et al. (17). This response profile suggests that the high expression of this gene may play a role in combating Mtb infection (31, 46). *GBP2* also participated in the cellular response to TNF. Marinho et al. (2020) (43) observed that mice deficient in GBP (GBP−/−) were more susceptible to Mtb infection, exhibiting a decreased expression of genes related to autophagy in the lungs in addition to a reduction in the production of pro inflammatory cytokines. TNF is also crucial in the formation and maintenance of granulomas. Changes in these cytokine levels have the ability to compromise the integrity of these structures, causing the reactivation of tuberculosis (47). In this context, it was concluded that *GBP2* is an important gene in stimulating cellular immunity and controlling mycobacterial infection (43). *GBP2* has also been associated with treatment monitoring (33) and its ability to discriminate active from latent TB (31).

As a final remark, the *C1QB* gene is involved in the regulation and activation pathways of the complement system, whose proteins participate in an innate and acquired defense mechanism by opsonizing pathogens and inducing inflammatory responses that help fight infections (36, 48). The high expression of this gene was also verified in the whole blood of patients with active tuberculosis in the Sambarey et al. study (2017) (49). However, in the present results, the *C1QB* gene obtained a significantly higher value of gene expression in the fluid of the non-TB group compared to the pleural tuberculosis group. In summary, the present results were similar to those described in a number of other works found in the literature (36, 48, 50).

When the CARD17, GBP2, and C1QB (**Figure 3B**) gene expressions in the blood of the non-TB group of patients following anti-tuberculosis therapy were checked, a reduction in the expression of all three genes was found. Activation of the *CARD17* gene triggers the activation of the innate immunity of the host during the inflammatory process as a result of its association with the inflammasome pathway during Mtb infection. The decline in the expression of this gene is indicative of patient cure and improvement in the inflammatory clinical picture, a finding corroborated by Natarajan et al. (2022) (30), demonstrating that the expression of *CARD17* in latent TB patients was significantly lower than in patients with active tuberculosis. The decrease in *GBP2* gene expression in the present cohort also suggests recovery of these tuberculous patients after treatment, as likewise observed in the works of Sambarey et al. (2017) (49) and Long et al. (2021) (33). As to the other genes, *C1QB* also showed a reduction in expression after tuberculosis treatment (49). Nonetheless, no significant *p*-value was observed in relation to the non-TB group.

Among the more favorable aspects of the present research, we highlight the following: (i) The originality of the study design. The investigation of the signature genes involved in the clinical form of PlTB, starting from previously established genes in the study of pulmonary tuberculosis, alludes to the greater reliability and discriminatory stability of these genes, making it possible to carry out gene expression assays by RT-qPCR, and thus, achieve our ultimate research objective. Moreover, until the completion of this work, we were not aware of the use of the same study design for the same purpose in the literature. (ii) The well-characterized study population in conformity with several standardized parameters provided by a tertiary care center in a highly TB-burdened country. Although any given study requires the meticulous application of the required eligibility criteria, our research was fundamentally based on two other previous PlTB studies performed by the present authors (5, 6). (iii) The relatively good sample size of our patient cohort suffering from this particular TB clinical form despite having to exclude samples from the volunteers who did not meet our eligibility and quality control criteria; and (iv) the ability to analyze paired samples in the post-treatment phase. At the same time, it is important to mention some of the major limitations of the present study such as a potential bias due to having to use transcriptomic datasets from pulmonary TB patients and not from a pleural tuberculosis cohort, which could have enriched the list of candidate genes related to a systemic or circulating (blood) response to the detriment of the genes that could have been expressed more explicitly at the infection site. However, our choice was justified by the absence of previously published transcriptomic datasets in pleural effusion samples.

In summary, we reanalyzed a previously published transcriptomic signature in pulmonary TB to identify candidate genes, which were measured and shown to discriminate PlTB from other causes of pleural effusion by using whole blood and PF. Among the top 10 genes, *CARD-17*, *GBP2*, and *C1QB* expressed in PF showed an above 80% accuracy rate in discriminating PlTB from other causes of pleural effusion.

## 5 Conclusion

Altogether, our findings presented new strategies in identifying diagnostic biomarkers in PlTB by using the previously known “*omics*” approaches, which, it is conjectured, could lead to a more accurate diagnosis and timely initiation of anti-TB therapy. Based on a reanalytical methodology by bioinformatics that utilizes a previously published transcriptomic public dataset in pulmonary tuberculosis, we succeeded in validating a total of three candidates, *CARD17, GBP2*, and *C1QB* genes, in clinical specimens. They distinguished themselves by showing promise in accurately discriminating PlTB from other causes of exudative pleural effusion, making it possible to reach a more reliable diagnosis and timely initiation of anti-tuberculosis therapy. In addition, in our view, our data provided a better understanding of the pathophysiological mechanisms of the disease, thereby making a decisive contribution to the development of new therapeutic methods and strategies in this important field.

## Data availability statement

The datasets presented in this study can be found in online repositories. The names of the repository/repositories and accession number(s) can be found in the article/**Supplementary Material**.

## Ethics statement

The studies involving humans were approved by Pedro Ernesto University Hospital (HUPE), Rio de Janeiro State University (UERJ). The studies were conducted in accordance with the local legislation and institutional requirements. The participants provided their written informed consent to participate in this study.

## Author contributions

RC: Formal Analysis, Investigation, Writing – original draft, Writing – review & editing, Data curation, Methodology, Validation, Visualization. TL-C: Data curation, Investigation, Methodology, Writing – original draft. TM: Investigation, Writing – original draft, Resources. AS: Investigation, Writing – original draft. JL: Investigation, Writing – original draft. RP: Writing – original draft, Writing – review & editing. RR: Writing – original draft, Formal Analysis, Visualization. MM: Conceptualization, Data curation, Formal Analysis, Writing – original draft. LR: Writing – original draft, Writing – review & editing, Conceptualization, Formal Analysis, Funding acquisition, Investigation, Project administration, Resources, Supervision.

## References

[1] Global tuberculosis report 2023 (2023). Available at: https://iris.who.int/.

[2] Global Tuberculosis reporT 2022 (2022). Available at: http://apps.who.int/bookorders.

[3] Governo do Estado do Rio de Janeiro (Brasil). Boletim Epidemiológico de Tuberculose. Secretaria de Saúde (2022). Available at: https://pesquisa.bvsalud.org/portal/resource/pt/biblio-1418663.

[4] Shaw JA Diacon AH Koegelenberg CFN. Tuberculous pleural effusion. Vol 24 Respirology Blackwell Publishing; (2019) p:962–71. doi: 10.1111/resp.13673 31418985

[5] Santos AP da Silva Corrêa R Ribeiro-Alves M da Silva ACOS Mafort TT Leung J. Application of Venn’s diagram in the diagnosis of pleural tuberculosis using IFN-γ, IP-10 and adenosine deaminase. PloS One (2018) 13(8):e0202481. doi: 10.1371/journal.pone.0202481 30148839 PMC6110466

[6] da Cunha Lisboa V Ribeiro-Alves M da Silva Corrêa R Ramos Lopes I Mafort TT Santos AP. Predominance of th1 immune response in pleural effusion of patients with tuberculosis among other exudative etiologies. J Clin Microbiol (2019) 58(1):e00927–19. doi: 10.1128/JCM.00927-19 PMC6935897 31619524

[7] Sharma SK Mitra DK Balamurugan A Pandey RM Mehra NK. Cytokine polarization in miliary and pleural tuberculosis. J Clin Immunol (2002) 22(6):345–52. doi: 10.1023/a:1020604331886 12462334

[8] Mitra DK Sharma SK Dinda AK Bindra MS Madan B Ghosh B. Polarized helper T cells in tubercular pleural effusion: Phenotypic identity and selective recruitment. Eur J Immunol (2005) 35(8):2367–75. doi: 10.1002/eji.200525977 16025563

[9] Rossi GA Balbi B Manca F. Tuberculous Pleural Effusions Evidence for selective Presence of PPD-Specific T-Lymphocytes at Site of Inflammation in the Early Phase of the Infection. Am Rev Respir Dis (1987) 136(3):575–9. doi: 10.1164/ajrccm/136.3.575 2443047

[10] Ramilo O Allman W Chung W Mejias A Ardura M Glaser C. Gene expression patterns in blood leukocytes discriminate patients with acute infections. Blood (2007) 109:2066–77. doi: 10.1182/blood-2006-02-002477 PMC1801073 17105821

[11] Ardura MI Banchereau R Mejias A Di Pucchio T Glaser C Allantaz F. Enhanced monocyte response and decreased central memory T cells in children with invasive Staphylococcus aureus infections. PloS One (2009) 4(5):e5446. doi: 10.1371/journal.pone.0005446 19424507 PMC2676512

[12] Guerreiro LTA Robottom-Ferreira AB Ribeiro-Alves M Toledo-Pinto TG Rosa Brito T Rosa PS. Gene expression profiling specifies chemokine, mitochondrial and lipid metabolism signatures in leprosy. PloS One (2013) 8(6):e64748. doi: 10.1371/journal.pone.0064748 23798993 PMC3683049

[13] Berry MPR Graham CM McNab FW Xu Z Bloch SAA Oni T. An interferon-inducible neutrophil-driven blood transcriptional signature in human tuberculosis. Nat (2010) 466(7309):973–7. doi: 10.1038/nature09247 PMC3492754 20725040

[14] Lesho E Forestiero FJ Hirata MH Hirata RD Cecon L Melo FF. Transcriptional responses of host peripheral blood cells to tuberculosis infection. Tuberculosis (2011) 91(5):390–9. doi: 10.1016/j.tube.2011.07.002 21835698

[15] Maertzdorf J Weiner J Mollenkopf HJ Network T Bauer T Prasse A. Common patterns and disease-related signatures in tuberculosis and sarcoidosis. Proc Natl Acad Sci U S A (2012) 109(20):7853–8. doi: 10.1073/pnas.1121072109 PMC3356621 22547807

[16] Ottenhoff THM Dass RH Yang N Zhang MM Wong HEE Sahiratmadja E. Genome-wide expression profiling identifies type 1 interferon response pathways in active tuberculosis. PloS One (2012) 7(9):e45839. doi: 10.1371/journal.pone.0045839 23029268 PMC3448682

[17] Zak DE Penn-Nicholson A Scriba TJ Thompson E Suliman S Amon LM. A blood RNA signature for tuberculosis disease risk: a prospective cohort study. Lancet (2016) 387(10035):2312–22. doi: 10.1016/S0140-6736(15)01316-1 PMC5392204 27017310

[18] Blankley S Graham CM Turner J Berry MPR Bloom CI Xu Z. The transcriptional signature of active tuberculosis reflects symptom status in extra-pulmonary and pulmonary tuberculosis. PloS One (2016) 11(10):e0162220. doi: 10.1371/journal.pone.0162220 27706152 PMC5051928

[19] Roe JK Thomas N Gil E Best K Tsaliki E MorrisJones S. Blood transcriptomic diagnosis of pulmonary and extrapulmonary tuberculosis. JCI Insight (2016) 1(16):e87238. doi: 10.1172/jci.insight.87238 27734027 PMC5053151

[20] D’Attilio L Díaz A Santucci N Bongiovanni B Gardeñez W Marchesini M. Levels of inflammatory cytokines, adrenal steroids, and mRNA for GRα, GRβ and 11βHSD1 in TB pleurisy. Tuberculosis (2013) 93(6):635–41. doi: 10.1016/j.tube.2013.07.008 23988280

[21] Espósito DLA Bollela VR Feitosa ALP da Fonseca BAL. Expression profiles of cytokine mRNAs in the pleural fluid reveal differences among tuberculosis, Malignancies, and pneumonia-exudative pleural effusions. Lung (2015) 193(6):1001–7. doi: 10.1007/s00408-015-9809-4 26407584

[22] Bloom CI Graham CM Berry MPR Rozakeas F Redford PS Wang Y. Transcriptional blood signatures distinguish pulmonary tuberculosis, pulmonary sarcoidosis, pneumonias and lung cancers. PloS One (2013) 8(8):e70630. doi: 10.1371/annotation/7d9ec449-aee0-48fe-8111-0c110850c0c1 23940611 PMC3734176

[23] Smyth GK. Linear models and empirical bayes methods for assessing differential expression in microarray experiments. Stat Appl Genet Mol Biol (2004) 3(1):article3. doi: 10.2202/1544-6115.1027 16646809

[24] Ramakers C Ruijter JM Lekanne Deprez RH Moorman AFM. Assumption-free analysis of quantitative real-time polymerase chain reaction (PCR) data. Neurosci Lett (2003) 339(1):62–6. doi: 10.1016/S0304-3940(02)01423-4 12618301

[25] Ruijter JM Ramakers C Hoogaars WMH Karlen Y Bakker O van den hoff MJB. Amplification efficiency: Linking baseline and bias in the analysis of quantitative PCR data. Nucleic Acids Res (2009) 37(6):e45. doi: 10.1093/nar/gkp045 19237396 PMC2665230

[26] Benjaminit Y Hochberg Y. Controlling the false discovery rate: a practical and powerful approach to multiple testing. J R Statist Soc B (1995) 57(1):289–300. doi: 10.1111/j.2517-6161.1995.tb02031.x

[27] Youden WJ. Index for rating diagnostic tests. Cancer (1950) 3(1):32–5. doi: 10.1002/1097-0142(1950)3:1<32:aid-cncr2820030106>3.0.co;2-3 15405679

[28] Seiscento M Conde MB Dalcolmo MMP. Tuberculous pleural effusions. J Bras Pneumol. (2006) 32(4):S174–81. doi: 10.1590/S1806-37132006000900003 17273621

[29] Zumla A Raviglione M Hafner R Fordham von Reyn C. Tuberculosis. New Engl J Med (2013) 368(8):745–55. doi: 10.1056/NEJMra1200894 23425167

[30] Natarajan S Ranganathan M Hanna LE Tripathy S. Transcriptional profiling and deriving a seven-gene signature that discriminates active and latent tuberculosis: an integrative bioinformatics approach. Genes (Basel) (2022) 13(4):616. doi: 10.3390/genes13040616 35456421 PMC9032611

[31] Perumal P Abdullatif MB Garlant HN Honeyborne I Lipman M McHugh TD. Validation of differentially expressed immune biomarkers in latent and active tuberculosis by real-time PCR. Front Immunol (2021) 11:612564. doi: 10.3389/fimmu.2020.612564 33841389 PMC8029985

[32] Nogueira BMF Krishnan S Barreto-Duarte B Araújo-Pereira M Queiroz ATL Ellner JJ. Diagnostic biomarkers for active tuberculosis: progress and challenges. EMBO Mol Med (2022) 14(12):e14088. doi: 10.15252/emmm.202114088 36314872 PMC9728055

[33] Long NP Phat NK Yen NTH Park S Park Y Cho YS. A 10-gene biosignature of tuberculosis treatment monitoring and treatment outcome prediction. Tuberculosis (2021) 131:102138. doi: 10.1016/j.tube.2021.102138 34801869

[34] Kozel TR Burnham-Marusich AR. Point-of-care testing for infectious diseases: past, present, and future. J Clin Microbiol (2017) 55(8):2313–20. doi: 10.1128/JCM.00476-17 PMC5527409 28539345

[35] Acharya B Acharya A Gautam S Ghimire SP Mishra G Parajuli N. Advances in diagnosis of Tuberculosis: an update into molecular diagnosis of Mycobacterium tuberculosis. Mol Biol Rep (2020) 47(5):4065–75. doi: 10.1007/s11033-020-05413-7 32248381

[36] Chen LH Liu JF Lu Y He XY Zhang C Zhou HH. Complement C1q (C1qA, C1qB, and C1qC) may be a potential prognostic factor and an index of tumor microenvironment remodeling in osteosarcoma. Front Oncol (2021) 11. doi: 10.3389/fonc.2021.642144 PMC8166322 34079754

[37] Zhai K Lu Y Shi HZ. Tuberculous pleural effusion. J Thorac Dis (2016), E486–94. doi: 10.21037/jtd.2016.05.87 PMC4958858 27499981

[38] Hertz D Schneider B. Sex differences in tuberculosis. Semin Immunopathol (2019) 41:225–37. doi: 10.1007/s00281-018-0725-6 30361803

[39] (2016). junior cts.

[40] Silva DR Rabahi MF Sant’Anna CC Da Silva-Junior JLR Capone D Bombarda S. Diagnosis of tuberculosis: a consensus statement from the Brazilian Thoracic Association. Jornal Brasileiro Pneumologia (2021) 47(2):e20210054. doi: 10.36416/1806-3756/e20210054 PMC8332844 34008763

[41] Madamarandawala P Rajapakse S Gunasena B Madegedara D Magana-Arachchi D. A host blood transcriptional signature differentiates multi-drug/rifampin-resistant tuberculosis (MDR/RR-TB) from drug susceptible tuberculosis: a pilot study. Mol Biol Rep (2023) 50(4):3935–43. doi: 10.1007/s11033-023-08307-6 36749527

[42] Huang Y Xu W Zhou R. NLRP3 inflammasome activation and cell death. Cell Mol Immunol (2021) 18(9):2114–27. doi: 10.1038/s41423-021-00740-6 PMC8429580 34321623

[43] Marinho FV Fahel JS de Araujo ACVSC Diniz LTS Gomes MTR Resende DP. Guanylate binding proteins contained in the murine chromosome 3 are important to control mycobacterial infection. J Leukoc Biol (2020) 108(4):1279–91. doi: 10.1002/JLB.4MA0620-526RR 32620042

[44] Bocchino M Bellofiore B Matarese A Galati D Sanduzzi A. IFN-γ release assays in tuberculosis management in selected high-risk populations. Expert Rev Mol Diagnostics (2009) 9:165–77. doi: 10.1586/14737159.9.2.165 19298140

[45] Li Q Li J Tian J Zhu B Zhang Y Yang K. IL-17 and IFN-γ production in peripheral blood following BCG vaccination and Mycobacterium tuberculosis infection in human. Eur Rev Med Pharmacol Sci (2012) 16(14):2029–36. 23242733

[46] Shi T Huang L Zhou Y Tian J. Role of GBP1 in innate immunity and potential as a tuberculosis biomarker. Sci Rep (2022) 12(1):11097. doi: 10.1038/s41598-022-15482-2 35773466 PMC9247026

[47] Godfrey MS Friedman LN. Tuberculosis and biologic therapies: anti-tumor necrosis factor-α and beyond. Clinics Chest Med (2019) 40:721–39. doi: 10.1016/j.ccm.2019.07.003 31731980

[48] Yang H Che D Gu Y Cao D. Prognostic and immune-related value of complement C1Q (C1QA, C1QB, and C1QC) in skin cutaneous melanoma. Front Genet (2022) 13. doi: 10.3389/fgene.2022.940306 PMC9468976 36110204

[49] Sambarey A Devaprasad A Baloni P Mishra M Mohan A Tyagi P. Meta-analysis of host response networks identifies a common core in tuberculosis. NPJ Syst Biol Appl (2017) 3(4). doi: 10.1038/s41540-017-0005-4 PMC5445610 28649431

[50] Kemp SB Steele NG Carpenter ES Donahue KL Bushnell GG Morris AH. Pancreatic cancer is marked by complement-high blood monocytes and tumor-associated macrophages. Life Sci Alliance (2021) 4(6):1–17. doi: 10.26508/lsa.202000935 PMC8091600 33782087

