# Supplementary material for: Reanalysis and validation of the transcriptional pleural fluid signature in pleural tuberculosis
Source: Front Immunol. 2024 Jan 15;14:1256558. doi: 10.3389/fimmu.2023.1256558 (PMC10822927; doi:10.3389/fimmu.2023.1256558)
Supplement: Supplementary file 1 [file DataSheet_1.docx]

Supplementary Material

Reanalysis and validation of tuberculosis signature genes in patients with exsudative pleural fluid

**Raquel da Silva Corrêa, Thyago Leal-Calvo, Thiago Thomaz Mafort, Ana Paula Santos, Janaina Leung, Roberta Olmo Pinheiro, Rogério Rufino, Milton Ozório Moraes, Luciana Silva Rodrigues***

*** Correspondence:** Corresponding Author: [lrodrigues.uerj@gmail.com](mailto:lrodrigues.uerj@gmail.com)


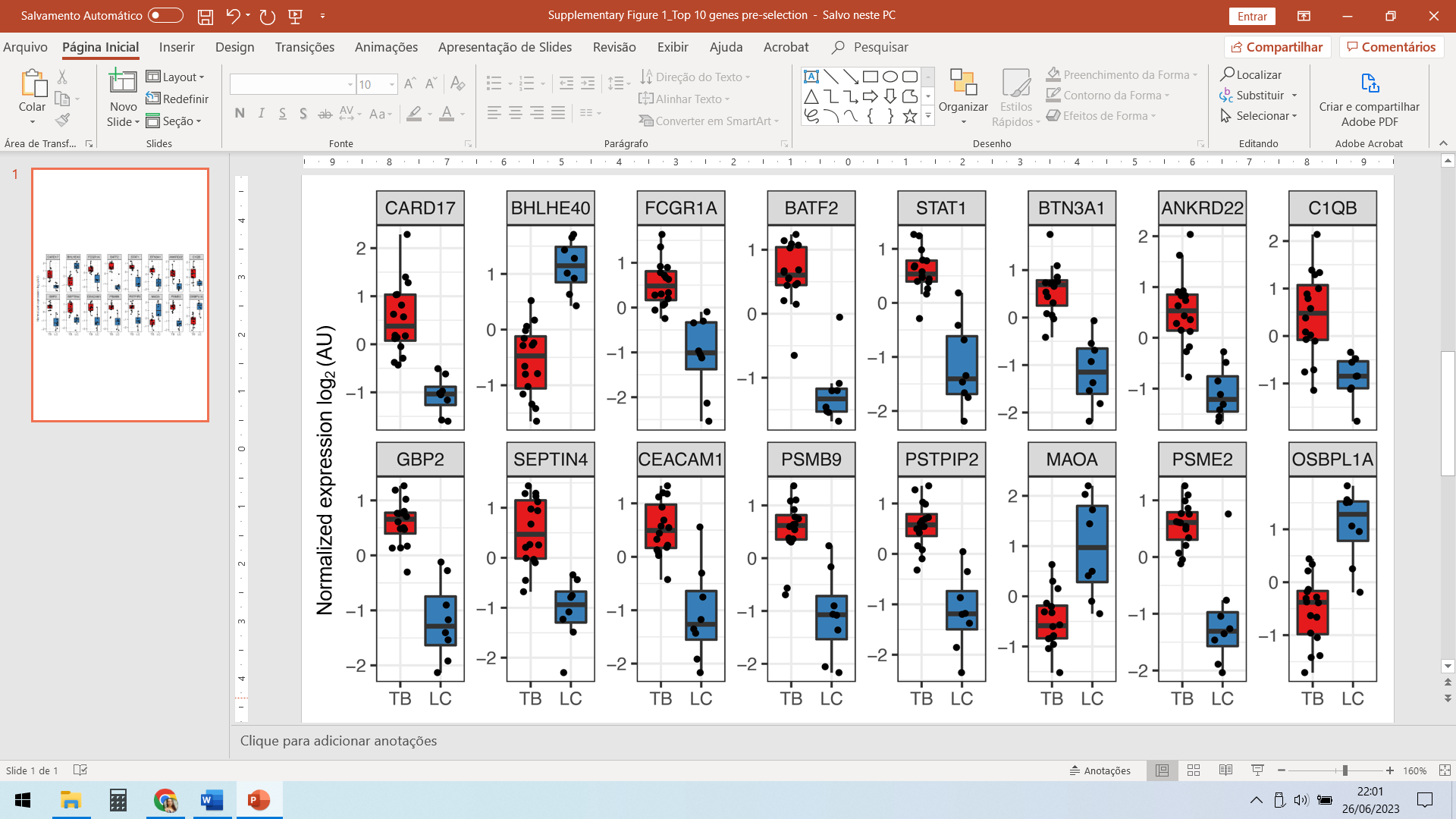


**Supplementary Figure 1:** Top 10 genes pre-selection. Expression of the 16 genes in the training set by microarray assays from whole blood samples from patients diagnosed with pulmonary tuberculosis and lung cancer from the study dataset by Bloom *et al* (2013). AU, arbitrary units; TB, pulmonary tuberculosis; LC, lung cancer.

**Supplementary Table 1:** Primers of genes selected by reanalysis. Ranking top 10 of the genes of interest and 2 reference genes obtained by reanalysis and synthesized with validation by gene expression by the RT-qPCR technique.

| **GENE** | **ENTREZ ID** | **TYPE** | **ORIENTATION** | **SEQUENCE 5’ – 3’** |
| --- | --- | --- | --- | --- |
| *CARD17* | 440068 | Interest | Forward | AATAGTACTTCCTTCCTAGGTTCA |
|  |  |  | Reverse | AGAGATAGAAACGTCTTGTCGAA |
| *BHLHE40* | 8553 | Interest | Forward | CATGTACCAAGTGTACAAGTCAA |
|  |  |  | Reverse | GGCAATTTGTAGGTCTCCTTG |
| *FCGR1A* | 2209 | Interest | Forward | GGGTTATACTGGTGCGAGGC |
|  |  |  | Reverse | AGTTGGTAACTGGAGGCCAAG |
| *BATF2* | 116071 | Interest | Forward | GCTGACCCAGACAGACC |
|  |  |  | Reverse | TTTCCAGAGACTCGTGCT |
| *STAT1* | 6772 | Interest | Forward | ATTCGACAGTATGATGAACACAGTATAG |
|  |  |  | Reverse | TCCCTAGAAACACAGGATGTGA |
| *BTN3A1* | 11119 | Interest | Forward | TCCAGAAGGAGATTTAACCATAG |
|  |  |  | Reverse | CCTTTGAGTCCCAACATTGAAA |
| *ANKRD22* | 118932 | Interest | Forward | CTGTAGAGGTGAAGTAAGCCTG |
|  |  |  | Reverse | TTGTCCAAAGTCATTCTGATAGG |
| *C1QB* | 713 | Interest | Forward | GTAGGCTCTCGGCTCCT |
|  |  |  | Reverse | GGATCTTCATCATCATACTGTGTC |
| *GBP2* | 2634 | Interest | Forward | CATGGACCAACTTCACTATGT |
|  |  |  | Reverse | GTCGTCTACAGAATTGTTACCAG |
| *SEPTIN4 (1°)* | 5414 | Interest | Forward | CTGGTCTTCTCTGACTCTCTG |
|  |  |  | Reverse | TGAGAAATCCTTCACGAACTTG |
| *SEPTIN4 (2°)* | 5414 | Interest | Forward | GACTCTCTGGAGATCAAGC |
|  |  |  | Reverse | CTGAGAAATCCTTCACGAACT |
| *RPLP2* | 6181 | Constitutive | Forward | CGCCAAGGACATCAAGAAG |
|  |  |  | Reverse | TCCATTCAGCTCACTGATAAC |
| *POLR2A* | 5430 | Constitutive | Forward | CGCATTGACTTGCGTTTCCA |
|  |  |  | Reverse | TGCCGTTCCACCTTATAGCC |

**Supplementary Table 2:** Classification of the top 10 genes by importance obtained by reanalysis. AUC, area under the curve; CI, confidence interval.

| **RANK** | **IMPORTANCE** | **GENE** | **AUC [95% CI]** | **Specificity** | **Sensitivity** |
| --- | --- | --- | --- | --- | --- |
| 1 | 100 | *CARD17* | 0.9 [0.75 - 1] | 0.75 | 1.00 |
| 2 | 75.988 | *BHLHE40* | 0.93 [0.82 - 1] | 0.75 | 1.00 |
| 3 | 71.429 | *FCGR1A* | 0.94 [0.85 - 1] | 1.00 | 0.82 |
| 4 | 61.097 | *BATF2* | 0.9 [0.7 - 1] | 0.88 | 1.00 |
| 5 | 49.331 | *BTN3A1* | 0.88 [0.67 - 1] | 0.88 | 0.91 |
| 6 | 47.385 | *C1QB* | 0.67 [0.41 - 0.93] | 1.00 | 0.45 |
| 7 | 46.259 | *ANKRD22* | 0.98 [0.92 - 1] | 1.00 | 0.91 |
| 8 | 40.893 | *GBP2* | 0.98 [0.92 - 1] | 1.00 | 0.91 |
| 9 | 39.337 | *STAT1* | 0.91 [0.73 - 1] | 0.88 | 1.00 |
| 10 | 36.937 | *SEPTIN4* | 0.78 [0.55 - 1] | 0.75 | 0.82 |
